# Penetrating abdominal injuries: management controversies

**DOI:** 10.1186/1757-7241-17-19

**Published:** 2009-04-17

**Authors:** Muhammad U Butt, Nikolaos Zacharias, George C Velmahos

**Affiliations:** 1 Division of Trauma, Emergency Surgery and Surgical Critical Care, Massachusetts General Hospital and Harvard Medical School, Boston, MA, USA

## Abstract

Penetrating abdominal injuries have been traditionally managed by routine laparotomy. New understanding of trajectories, potential for organ injury, and correlation with advanced radiographic imaging has allowed a shift towards non-operative management of appropriate cases. Although a selective approach has been established for stab wounds, the management of abdominal gunshot wounds remains a matter of controversy. In this chapter we describe the rationale and methodology of selecting patients for non-operative management. We also discuss additional controversial issues, as related to antibiotic prophylaxis, management of asymptomatic thoracoabdominal injuries, and the use of colostomy vs. primary repair for colon injuries.

## Introduction

Penetrating trauma of the abdomen continues to be a major cause of trauma admission in the United States. Stab wounds (SW) are encountered three times more often than gunshot wounds (GSW), but have a lower mortality because of the lower energy transmitted. Approximately 90% of the deaths related to penetrating abdominal injury (PAI) are caused by GSW. Prior to World War I, PAI was managed expectantly. During World War II, however, studies showed that early laparotomy improved survival. By the late 1950s, routine laparotomy was the standard treatment for PAI. Over the last 30 years the pendulum shifted towards selective management, initially involving only SW and later including GSW. The introduction and refinement of diagnostic procedures and imaging studies, such as laparoscopy, computed tomographic (CT) scan, and focused abdominal sonography for trauma (FAST), has contributed significantly in the new trends of PAI management.

In this article we discuss the main controversies of PAI care. For convenience we have divided them into five categories.

### A. Selective Non-Operative Management of SW

Patients are selected for non-operative management based on the absence of hemodynamic instability and peritonitis. Both of these terms are relative and a single value is inappropriate to define them. Although a systolic blood pressure value of 90 mmHg or lessand a heart rate of 100 beats/minute or more are considered hypotension and tachycardia (and therefore denote hemodynamic instability), patients can be in shock with values that are seemingly normal. Age, physiologic conditioning, co-morbid conditions, and medications are some of the factors that may affect blood pressure and heart rate, providing misleading information, if one were to rely only on absolute values. Similarly, peritonitis is determined by the individual patient's threshold of pain, and this is vastly different. Therefore, the astute clinician needs to monitor the patient carefully, assess the situation as a whole picture and not as fragmented informational pieces, and determine the presence of hemodynamic instability or peritonitis based on knowledge, experience, and the ability to practice the art of surgery.

Trauma surgeons who routinely manage penetrating trauma may feel more comfortable dealing with a selective non-operative approach. A lower threshold for surgical exploration is not unreasonable for physicians who do not treat such patients frequently. On the other hand, the principles of hemodynamic stability, diffuse abdominal tenderness, close monitoring, and repeat clinical exams are similar across specialties or types of patients. If the local hospital infrastructure allows these principles to be applied, physicians should be able to safely select those patients that do not require an unnecessary operation.

**Figure 1:**
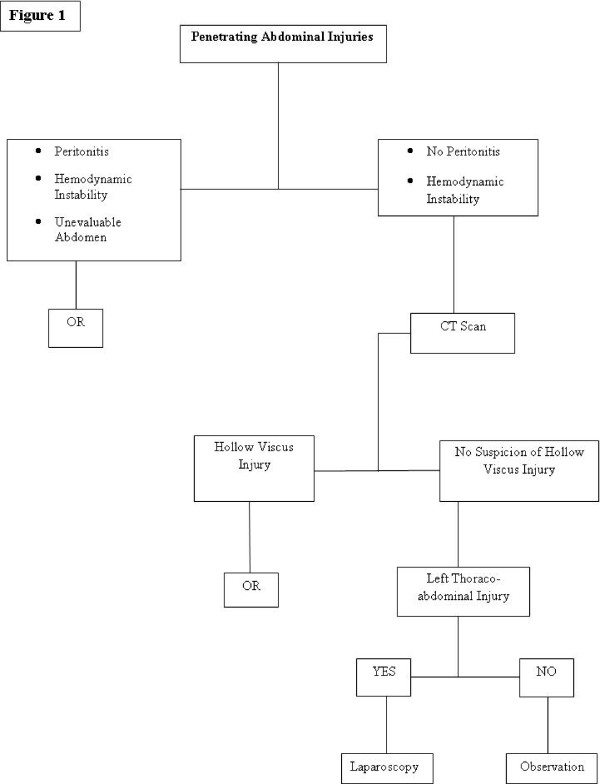
**Algorithm for Management of Penetrating Abdominal Injuries**.

Close monitoring and follow-up are mandatory in patients managed non-operatively. These patients should have repeat clinical exams -by preferably the same physician- over the 12–24 hours ensuing arrival to the hospital. Observation periods over 24 hours are rarely required (Figure 1).

#### 1. Anterior Abdomen

Selective non-operative management (SNOM) of abdominal SW has become the standard of care in the United States. About 55% of stab wounds to the anterior abdomen can safely be managed non-operatively [1]. Even in the presence of peritoneal violation, a significant number of patients have no major intra-abdominal injury requiring an operation.

The first report of SNOM of penetrating injuries to the liver was published in 1986. In this prospective study of 63 SW patients Demetriades et al showed that 33% of the time the liver was successfully managed nonoperatively [2].

In a prospective study of 651 patients with SW, Demetriades et al demonstrated that, based on initial and serial clinical examination, 306 (47%) of the patients could be managed conservatively [3]. Of the 651 patients, 345 (53%) had signs of an acute abdomen and were operated on immediately. The remaining 306 patients had minimal or no peritoneal signs and were observed. This group included 26 patients with omental or intestinal evisceration, 18 patients with air under the diaphragm, 12 patients with blood on abdominal paracentesis, and 18 patients with shock on admission. The authors stressed that none of the above signs were an absolute criterion for mandatory exploration and that SNOM could be offered, even if with a heightened index of suspicion for injury and lowered threshold for an operation.

In a more recent report, Demetriades et al conducted a prospective study of 152 patients with penetrating injuries to abdominal solid viscera [4]. Forty-five patients (29.6%) were stabbed. The liver was the most commonly injured solid organ (73%), followed by the kidney (30.3%) and the spleen (30.3%). Forty-one patients (27%), including 18 cases with grade III to V injuries, were successfully managed without a laparotomy and without any abdominal complication. Patients with isolated solid organ injuries treated non-operatively had a significantly shorter hospital stay than patients treated operatively (mean, 3 days vs. 6 days respectively, p = 0.002), even though the former group had more severe injuries. The authors concluded that in the appropriate environment, SNOM of penetrating abdominal solid organ injuries has a high success rate and a low complication rate. The appropriate environment constituted a trauma center with a dedicated in-house team that could monitor and serially examine the patient during the observation period.

Similarly in another prospective randomized study by Leppaniemi et al. 51 patients with stab wounds to the anterior abdomen that did not require immediate laparotomy were assigned to nonoperative management or mandatory laparotomy. In this study the authors looked at the safety and cost-effectiveness of both management techniques. The morbidity rate was 19% following mandatory laparotomy and 8% after observation (p = 0.26). The hospital stay was shorter in the observation group (median 2 days vs. 5 days; p = 0.002). About $2800 (US) was saved for every patient who underwent successful nonoperative management. The authors concluded that selective nonoperative management of abdominal stab wounds, although resulting in delayed laparotomy in some patients, is safe and the preferred strategy for minimizing the days in hospital as well as hospital costs [5].

#### 2. Posterior Abdomen (back)

Penetrating injuries of the back are generally considered as a distinct form of abdominal trauma. The back can be defined as the area between the tips of the scapulae, the iliac crests, and both midaxillary lines. The vertebral column and the heavy paraspinous musculature provide a better protective barrier than the anterior abdominal wall, and retroperitoneal injuries may not be clinically detectable in the early stages. Missed colonic or duodenal perforations, urinary tract and vascular wounds may have devastating outcomes. For these reasons many have raised concerns about the safety of SNOM of penetrating injuries of the back.

Demeteriades et al in a prospective study of 230 patients with penetrating injuries to the back showed that195 patients were managed without requiring an operation, 30 underwent a therapeutic and 5 a non-therapeutic laparotomy. Ninety-seven percent of the patients had SW as the mechanism of injury [6]. The diagnostic accuracy of the initial abdominal examination was 95.2% (false positive: 2.6%; false negative: 2.2%). Peritoneal lavage was mentioned only to be condemned. The value of plain chest X-ray was very limited with a positive yield of 2.6%. Intavenous pyelograms were performed only for wounds over the kidneys with gross hematuria or 4+ hematuria on dipstick. The author concluded that penetrating injuries of the back should be assessed in the same way as those of the anterior abdomen.

In a large series of 465 patients with penetrating stab wounds to the back, Peck and Berne showed that physical examination was extremely reliable in deciding when to do a laparotomy [7]. Eighty percent of the patients were managed non-operatively. Tenderness not localized to the area of injury and absent or rare bowel sounds best identified patients with serious injuries. Again peritoneal lavage was used infrequently. Colon, vascular and diaphragmatic injuries were more common. The flank was more vulnerable than the back. They concluded that SNOM for all patients with penetrating stab wounds to the back was a reliable and prudent approach.

Therefore, in the absence of obvious signs of significant organ or vascular injury, the best management plan for patients with stab wounds of the posterior abdomen is SNOM. Repeated physical exams are the mainstay of treatment, as indicated by many authors. Other studies, such as diagnostic peritoneal lavage, angiography, intravenous pyelography, and contrast CT scanning, are indicated on a case-by-case basis, but as yet lack convincing justification for routine use [8].

### B. Selective Non-Operative Management of GSW

#### 1. Anterior Abdomen

Despite initial disbelief, SNOM of anterior abdominal GSW has gained significant momentum and is widely used, particularly by experienced trauma surgeons.

Patients selected for SNOM are hemodynamically stable, without diffuse abdominal tenderness, and with a reliable clinical examination. Patients who are hemodynamically unstable, with diffuse abdominal tenderness or having associated head injuries, spinal cord injury, and severe intoxication should be almost always explored. SNOM patients are observed and closely monitored for at least 12–24 hours. During the observation period, serial abdominal exams are performed, preferably by the same physician, and additional radiologic tests may be ordered with a CT scan being the most common diagnostic adjunct.

SNOM of abdominal gunshot wounds was first introduced by Shaftan in the 1960s, presenting the idea of "selective conservativism" [9]. Two larger studies followed validating this idea by the same group and by Nance et al. [10,11].

In a study published in 2001 by Velmahos et al 1,975 patients with abdominal GSW were included and 1,405 of them had anterior abdominal GSW; 484 patients were managed non-operatively (34%) [12]. Sixty-five patients received a delayed laparotomy (13.4%) after developing signs or symptoms but only 48 (9.9%) had significant injuries. Seventeen patients had a non-therapeutic laparotomy (26.2%).

In a similar study published earlier by the same group, 106 patients with anterior abdominal GSW were managed by SNOM and the success rate was 86.8% [13].

FAST can be a useful initial diagnostic study after PAI but due to its low sensitivity it cannot be relied upon for distinguishing PAI patients who may or may not need surgical exploration. In a prospective study by Udofi et al. out of 75 patients with PAI, 54 had a negative FAST. 13 out of the 54 had a false negative FAST and on further evaluation had significant organ injury. This resulted in the test having a sensitivity of 46% [14].

SNOM patients can be reliably evaluated with an abdominal CT scan [15]. Significant intraabdominal injuries and the bullet trajectory can be identified with a sensitivity and specificity of >90% [15]. Although careful and repeat clinical examination is the mainstay for non-operative management, CT scan can be a useful adjunct in identifying patients for operative management. Similar results were reported by Grossman et al. and Munera et al. with a low rate of negative laparotomy of 9–19% [16,17].

New high-quality helical CT scans with the ability to offer multiplanar reconstructions have assisted greatly in establishing the trajectory in gunshot wounds of the torso. The CT scan becomes particularly useful, if it can determine a trajectory that is confirmatively outside the peritoneal cavity. Then, such patients can be discharged immediately following the CT scan. On all other occasions, the patient should either be closely observed (undetermined trajectory) or operated on (trajectory causing clinically significant injuries).

#### 2. Back and buttocks

The management of GSW of the back and buttocks follows the same principles with anterior abdomen GSW.

Velmahos et al in a prospective study evaluated 192 patients with GSW to the back over a 12 month period [18]. One hundred and thirty patients with initial negative examination were selected for SNOM. Four patients received a delayed laparotomy but it was non-therapeutic in all of them. SNOM was successful in the remaining 126 patients (96.9%). The clinical examination had a sensitivity of 100% and specificity of 95%. The observation period remains approximately 12–24 hours. The same group published a study regarding management of GSW to the buttocks with potential trajectories to the retroperitoneum, reaching the conclusion that clinical examination is a reliable predictor for the need of an operation [19]. A rigid sigmoidoscopy was introduced per routine in all cases.

#### 3. Transpelvic GSW

Even if, in previous times, it would be almost impossible to believe that a transpelvic GSW could have been managed by SNOM, Velmahos et al have shown that the principle of guiding surgical intervention on the basis of a good clinical exam and appropriate imaging tests is valid even in this scenario. Of 37 patients with transpelvic gunshot wounds, who were collected during a 12-year period [20], the authors managed 18 patients by NOM. Three received a delayed laparotomy; in all cases non-therapeutic. Clinical examination carried a sensitivity of 100% and a specificity of 71.4%.

### C. Management of Asymptomatic Left Thoraco-abdominal Penetrating Injuries

Patients with penetrating injuries to the left thoraco-abdominal area are at high risk of diaphragmatic injury. The left thoracoabdominal region is defined by the nipple line superiorly and the costal margin inferiorly. The medial margins are the sternum anteriorly and the spine posteriorly.

Murray et al prospectively collected patients with penetrating left thoracoabdominal wounds [21]. The incidence of diaphragmatic injuries in GSW patients was 59% (23 of 39). Patients with indications for surgery (peritonitis, hemodynamic instability, hemothorax, and cardiac tamponade) underwent exploration, whereas patients with no indications underwent laparoscopy. Among patients evaluated laparoscopically, 10% were found to have diaphragmatic injuries. Whereas, among patients explored, 76% were found to have a diaphragmatic injury. In a study by the same group, patients with penetrating thoraco-abdominal injuries were evaluated by laparoscopy for diaphragmatic injuries. The incidence of occult diaphragmatic injuries was 24% [22].

The natural history of unrepaired diaphragmatic lacerations is unknown. Although large lacerations may cause intra-thoracic herniation and visceral strangulation, smaller lacerations most likely heal or are sealed by omentum. For patients without an indication for laparotomy, laparoscopy is considered a reasonable alternative to rule out diaphragmatic injuries, particularly if a larger than 2 cm laceration is suspected. The excellent accuracy of CT (96%) to detect diaphragmatic injuries, as shown by the Maryland group, has not been duplicated by others. The groups conclude that with increasingly evolving technology, CT may become the standard of care for identifying such injuries [23].

### D. Primary Repair vs. Colostomy for Colon Injuries

The management of colon injuries has undergone major changes in the last three decades. After the World War II era all colonic injuries were routinely managed by performing a colostomy. The policy of mandatory colostomy was carried on until the 1970's. This was gradually replaced by primary repair in selected cases in the late 1970s and by liberal primary repair in most cases in the 1990's.

The first strong study to challenge the mandatory colostomy practice was made by Stone and Fabian in 1979 [24]. In a prospective randomized study of 268 patients they showed that in selected patients primary repair was associated with fewer complications than colostomy (15% vs. 29%, p < 0.05). The authors concluded that patients that satisfied the specific criteria of, 1) preoperative shock never being profound; 2) blood loss less than 20% of estimated normal volume; 3) no more than two intra-abdominal organs injured; 4) minimal fecal contamination; 5) operation within eight hours; and, 6) wounds of colon and abdominal wall never so destructive as to require resection, should have primary closure as the preferred method of treatment.

Gonzalez et al randomized 109 patients with colon injuries to a primary repair group or a diversion group, independent of any risk factors [25]. The sepsis-related complication rate was 20% in the primary repair group versus 25% in the diversion group, although this was not significant. The authors concluded that all penetrating colon injuries, including those requiring resection, should be primarily repaired.

Cornwell et al in their prospective study [26] and a couple of retrospective studies, that included a large number of colon injuries requiring resection, advocate a cautious approach and support diversion in the presence of certain risk factors, such as Penetrating Abdominal Trauma Index > 25 (PATI), multiple blood transfusions, hypotension, or underlying medical illnesses.

Nelson et al. in their meta-analysis study of randomized controlled trials of primary repair vs. fecal diversion for penetrating colon injuries showed that there was no difference in mortality between the two groups (OR 1.70, 95% CI 0.51 – 5.66). However, total complications (OR 0.28 95% CI 0.18 – 0.42), total infectious complications (OR 0.41, 95% CI 0.27 – 0.63), abdominal infections including dehiscence (OR 0.59, 95% CI 0.38 – 0.94), abdominal infections excluding dehiscence (OR 0.52 95% CI 0.31 – 0.86), wound complications including dehiscence (OR 0.55, 95% CI 0.34 – 0.89), and wound complications excluding dehiscence (OR 0.43, 95% CI 0.25 – 0.76) all significantly favored primary repair. The authors concluded that the currently published randomized controlled trials favors primary repair over fecal diversion for penetrating colon injuries [27].

In a recent landmark prospective multicenter study of 297 patients, Demetriades et al compared primary anastomosis with diversion [28]. There were 197 patients in the primary anastomosis group as compared to the 100 in the diversion group. There was no difference in colon-related complications between the two groups (22% vs. 27%, p < 0.313 for primary anastomosis and diversion respectively). Multivariate analysis including all potential risk factors with p values < 0.2 identified three independent risk factors for abdominal complications: severe fecal contamination, transfusion of > 3 units of blood within the first 24 hours, and single-agent antibiotic prophylaxis. The type of colon management was not found to be a risk factor. The authors concluded that in severe colon injuries requiring resection, the method of colon management does not influence the incidence of colon-related abdominal complications, irrespective of the presence or absence of any risk factors. The intensive care unit and hospital stays were shorter in the primary repair group, although not statistically significantly. In view of these findings and the fact that colon diversion is associated with worse quality of life and requires an additional operation for closure, colon injuries requiring resection should be managed by primary repair, irrespective of risk factors.

### E. Prophylactic Antibiotics for Penetrating Abdominal Injuries

Presumptive antibiotic therapy is administered in PAI to reduce the incidence of postoperative infection. However, the appropriate timing, duration, and choice of antibiotics are still a matter of debate, although most clinicians lean towards a single-broad agent administered over 24 hours post-operatively for most of the cases.

Fabian et al conducted a prospective double blind study of 515 PAI patients that received either 2 g cefoxitin or cefotetan for 24 hours or 5 days [29]. Major abdominal infections (MAI) were defined as abscesses, necrotizing fasciitis, and diffuse peritonitis. There were no statistically significant differences in MAI between the two groups (MAI of Colon, 24 hour, 14%; 5 days, 15%). They concluded that regardless of contamination and degree of injury, 24 hour antibiotic therapy is satisfactory for all PAI patients.

In a similar study Bozorgzadeh et al randomized 300 PAI patients to receive 1 gm intravenous cefoxitin (instead of the conventional 2 gm) for 24 hours or 5 days [30]. There was again no postoperative mortality, and no differences in overall length of hospitalization between the two groups. The duration of antibiotic treatment had no influence on the development of any infection (p = 0.136) or an intra-abdominal infection (p = 0.336). Only colon injury was an independent predictor of the development of an intra-abdominal infection (p = 0.0031). The authors concluded that 24 hours of intravenous cefoxitin vs. 5 days of therapy made no difference in the prevention of postoperative infection or length of hospitalization.

In another prospective randomized study Cornwell et al treated 63 high risk patients (defined as PAI with full thickness colon injury and one of the following: (1) PATI > 25, (2) transfusion of 6 units or more of packed red blood cells, or (3) > 4 hours from injury to operation.) with 24 hour vs. 5 day of 2 gm cefoxitin [31]. The study showed that there was no statistically significant differences in intra-abdominal (24 hour, 19%; 5-days, 38%) and extra-abdominal (24 hour, 45%; 5-days, 25%) infection rates between the two groups. The authors concluded that even in the highest risk PAI patients, extending prophylactic antibiotics to more than 24 hours is of no benefit.

## Summary

The field of PAI care has drastically changed over the last few decades. SNOM has become the standard of care for SW and is increasingly gaining acceptance for GSW. Non-operatively managed patients are usually observed for 12–24 hours. CT scan is used frequently to help determine the need for operation and has replaced other tests, such as diagnostic peritoneal lavage or intravenous pyelography. Despite FAST being a very useful tool in the evaluation of blunt trauma patients, it cannot be relied upon for distinguishing PAI patients who may or may not need surgical exploration. Penetrating colon injuries are almost always managed by primary repair. A single broad-spectrum antibiotic agent administered over 24 hours is as good for prophylaxis against infection as longer regimens in PAI patients.

## Competing interests

The authors declare that they have no competing interests.

## Authors' contributions

MUB contributed to section A, B and E of the review. NZ contributed to section C and D of the review. GCV contributed to the whole review, and drafted the manuscript. All authors read and approved the final manuscript.

## References

[B1] Navsaria PH, Berli JU, Edu S, Nicol AJ (2007). Non-operative management of abdominal stab wounds – an analysis of 186 patients. S Afr J Surg.

[B2] Demetriades D, Rabinowitz B, Sofianos C (1986). Non-operative management of penetrating liver injuries: a prospective study. Br J Surg.

[B3] Demetriades D, Rabinowitz B (1987). Indications for operation in abdominal stab wounds. A prospective study of 651 patients. Ann Surg.

[B4] Demetriades D, Hadjizacharia P, Constantinou C, Brown C, Inaba K, Rhee P, Salim A (2006). Selective nonoperative management of penetrating abdominal solid organ injuries. Ann Surg.

[B5] Leppäniemi AK, Haapiainen RK (1996). Selective nonoperative management of abdominal stab wounds: prospective, randomized study. World J Surg.

[B6] Demetriades D, Rabinowitz B, Sofianos C, Charalambides D, Melissas J, Hatzitheofilou C, Da Silva J (1988). The management of penetrating injuries of the back. A prospective study of 230 patients. Ann Surg.

[B7] Peck JJ, Berne TV (1981). Posterior abdominal stab wounds. J Trauma.

[B8] Berne TV (1990). Management of penetrating back trauma. Surg Clin North Am.

[B9] Shaftan GW (1969). Selective conservativism in penetrating abdominal trauma. J Trauma.

[B10] McAlvanah MJ, Shaftan GW (1978). Selective conservativism in penetrating abdominal wounds: a continuous reppraisal. J Trauma.

[B11] Nance FC, Wennar MH, Johnson LW, Ingram JC, Cohn I (1974). Surgical judgement in the management of penetrating wounds of the abdomen: Experience with 2,212 patients. Ann Surg.

[B12] Velmahos GC, Demetriades D, Toutouzas KG, Sarkisyan G, Chan LS, Ishak R, Alo K, Vassiliu P, Murray JA, Salim A, Asensio J, Belzberg H, Katkhouda N, Berne TV (2001). Selective nonoperative management in 1,856 patients with abdominal gunshot wounds: should routine laparotomy still be the standard of care?. Ann Surg.

[B13] Demetriades D, Velmahos GC, Cornwell E, Berne TV, Cober S, Bhasin PS, Belzberg H, Asensio J (1997). Selective nonoperative management of gunshot wounds to the anterior abdomen. Arch Surg.

[B14] Udobi KF, Rodriguez A, Chiu WC, Scalea TM (2001). Role of ultrasonography in penetrating abdominal trauma: a prospective clinical study. J Trauma.

[B15] Velmahos GC, Constantinou C, Tillou A, Brown CV, Salim A, Demetriades D (2005). Abdominal Computed Tomographic Scan for Patients with Gunshot Wounds to the Abdomen Selected for nonoperative Management. J Trauma.

[B16] Grossman MD, May AK, Schwab CW, Reilly PM, McMahon DJ, Rotondo M, Shapiro MB, Kauder DR, Frankel H, Anderson H (1998). Determining anatomic injury with computed CT Tomography in selected torso gunshot wounds. J Trauma.

[B17] Múnera F, Morales C, Soto JA, Garcia HI, Suarez T, Garcia V, Corrales M, Velez G (2004). Gunshot wounds of abdomen: evaluation of stable patients with triple-contrast helical CT. Radiology.

[B18] Velmahos GC, Demetriades D, Foianini E, Tatevossian R, Cornwell EE, Asensio J, Belzberg H, Berne TV (1997). A selective approach to the management on gunshot wounds to the back. Am J Surg.

[B19] Velmahos GC, Demetriades D, Cornwell EE, Asensio J, Belzberg H, Berne TV (1997). Gunshot wounds to the buttocks: predicting the need for operation. Dis Colon Rectum.

[B20] Velmahos GC, Demetriades D, Cornwell EE (1998). Transpelvic gunshot wounds: Routine laparotomy or selective management?. World J Surg.

[B21] Murray JA, Demetriades D, Cornwell EE, Asensio JA, Velmahos G, Belzberg H, Berne TV (1997). Penetrating left thoracoabdominal trauma: The incidence and clinical presentation of diaphragm injuries. J Trauma.

[B22] Murray JA, Demetriades D, Asensio JA, Cornwell EE, Velmahos GC, Belzberg H, Berne TV (1998). Occult Injuries to the Diaphragm: Prospective evaluation of laparoscopy in penetrating injuries to the left lower chest. JACS.

[B23] Stein DM, York GB, Boswell S, Shanmuganathan K, Haan JM, Scalea TM (2007). Accuracy of computed tomography (CT) scan in the detection of penetrating diaphragm injury. J Trauma.

[B24] Stone HH, Fabian TC (1979). Management of perforating colon trauma: randomization between primary closure and exteriorization. Ann Surg.

[B25] Gonzalez RP, Merlotti GJ, Holevar MR (1996). Colostomy in penetrating colon injury: is it necessary?. J Trauma.

[B26] Cornwell EE, Velmahos GC, Berne TV, Murray JA, Chahwan S, Asensio J, Demetriades D (1998). The fate of colonic suture lines in high-risk trauma patients: a prospective analysis. J Am Coll Surg.

[B27] Nelson R, Singer M (2002). Primary repair for penetrating colon injuries. Cochrane Database Syst Rev.

[B28] Demetriades D, Murray JA, Chan L, Ordoñez C, Bowley D, Nagy KK, Cornwell EE, Velmahos GC, Muñoz N, Hatzitheofilou C, Schwab CW, Rodriguez A, Cornejo C, Davis KA, Namias N, Wisner DH, Ivatury RR, Moore EE, Acosta JA, Maull KI, Thomason MH, Spain DA (2001). Committee on Multicenter Clinical Trials. American Association for the Surgery of Trauma. Penetrating colon injuries requiring resection: diversion or primary anastomosis? An AAST prospective multicenter study. J Trauma.

[B29] Fabian TC, Croce MA, Payne LW, Minard G, Pritchard FE, Kudsk KA (1992). Duration of antibiotic therapy following penetrating abdominal trauma: a prospective trial. Surgery.

[B30] Bozorgzadeh A, Pizzi WF, Barie PS, Khaneja SC, LaMaute HR, Mandava N, Richards N, Noorollah H (1999). The duration of antibiotic administration in penetrating abdominal trauma. Am J Surg.

[B31] Cornwell EE, Dougherty WR, Berne TV, Velmahos G, Murray JA, Chahwan S, Belzberg H, Falabella A, Morales IR, Asensio J, Demetriades D (1999). Duration of antibiotic prophylaxis in high-risk patients with penetrating abdominal trauma: a prospective randomized trial. J Gastrointest Surg.

